# Human platelet interaction with *E*. *coli* O111 promotes tissue-factor-dependent procoagulant activity, involving Toll like receptor 4

**DOI:** 10.1371/journal.pone.0185431

**Published:** 2017-09-28

**Authors:** Valeria Matus, J. Guillermo Valenzuela, Patricia Hidalgo, L. María Pozo, Olga Panes, Aniela Wozniak, Diego Mezzano, Jaime Pereira, Claudia G. Sáez

**Affiliations:** 1 Department of Hematology-Oncology, School of Medicine, Pontificia Universidad Católica de Chile, Santiago, Chile; 2 Department of Clinical Laboratory, School of Medicine, Pontificia Universidad Católica de Chile, Santiago, Chile; Rutgers University, UNITED STATES

## Abstract

Platelets have a major role in clotting activation and contribute to the innate immune response during systemic infections. Human platelets contain tissue factor (TF) and express functional Toll-like receptor 4 (TLR4). However, the role of TLR4 in triggering the procoagulant properties of platelets, upon challenge with bacteria, is yet unknown. Our hypothesis is that *E*. *coli* O111-TLR4 interaction activates platelets and elicits their procoagulant activity. We demonstrated that the strain, but not ultrapure LPS, increased surface P-selectin expression, platelet dependent TF procoagulant activity (TF-PCA) and prompted a faster thrombin generation (TG). Blockade of TLR4 resulted in decreased platelet activation, TF-PCA and TG, revealing the participation of this immune receptor on the procoagulant response of platelets. Our results provide a novel mechanism by which individuals with bacterial infections would have an increased incidence of blood clots. Furthermore, the identification of platelet TF and TLR4 as regulators of the effect of *E*. *coli* O111 might represent a novel therapeutic target to reduce the devastating consequences of the hemostatic disorder during sepsis.

## Introduction

Platelets play a major function in hemostasis and thrombosis, but also have an active role in inflammatory and anti-microbial processes [1]. Amongst numerous surface receptors, human and murine platelets express functional Toll Like Receptors (TLRs) [2], which are important in innate and adaptive immunity [3], inflammation and atherosclerosis [4,5]. Specifically, TLR4 has a major role in sensing the lipopolysaccharide (LPS) of Gram-negative bacteria. This pathogen-associated molecular pattern (PAMP) is an early marker of bacterial invasion and initiator of the innate immune response [6,7]. However, when amplified and uncontrolled during infections, the subsequent inflammatory reaction may lead to septic shock syndrome [8]. Accordingly, advances in the knowledge of platelet-bacteria interaction may be clinically translated for preventing and treating severe bacterial infections [9].

*E*. *coli* is the most common cause of urinary tract infections in humans [10] and other enteric and systemic infections, including bacteremia [11] and neonatal meningitis [12], resulting in major clinical burden and thousands of deaths per year. *E*. *coli* endotoxins prompt the clinical expression of Gram-negative sepsis, i.e., fever, hypotension and disseminated intravascular coagulation. Enterohemorrhagic *E*. *coli* (EHEC) is pathogenically involved in the hemolytic uremic syndrome (HUS) [13] and the serotype O111 is particularly prevalent, causing outbreaks of the disease [14,15].

Platelets TLR4 response to pure *E*. *coli* LPS *in vitro* has not been unequivocally established[16]. *E*. *coli* LPS-exposed platelets result neither in increased CD62P surface expression nor in rise of cytosolic Ca^+2^ concentration [17–19]. Furthermore, this LPS does not prime platelet aggregation by sub-threshold concentrations of agonists, i.e., ADP or platelet-activating factor (PAF) [17,20]. Of note, recent reports in animal models would not accurately reflect the genetic and proteomic changes associated with inflammatory stress in humans [21,22]. In this regard, *ex vivo* studies using human platelets and live bacteria might be a plausible approach to mimic the platelet-bacteria interaction and, hence, to disclose mechanistic features in human infections.

Inflammation-induced clotting is triggered by tissue factor (TF) activation [23], and the thrombin generated stimulates cytokine production by leukocytes and endothelium, boosting further inflammation and inducing tissue cell proliferation and migration [24,25]. In this context, it is well known that severe infections are associated with increased platelet consumption and frequently with thrombocytopenia[26]. However, and although the understanding of the mechanisms involved in blood coagulation abnormalities in systemic infections has gradually progressed, the contribution of platelets to the clotting activation needs to be further explored.

Platelets contain TF mRNA, synthesize the protein, which is present in circulating platelets [27,28] and express TF activity upon agonist stimulation [29]. Rondina *et al*. [30] showed that live bacteria and bacterial toxins can directly induce expression of mature TF mRNA and synthesis of the protein by human platelets, contributing to cascades of procoagulant activity, fibrin deposition and pro-inflammatory signaling. However, the prothrombotic consequences of platelet TLR4 stimulation with a live Gram-negative, such as EHEC, are mostly unknown.

Accordingly, this study assesses the *ex vivo* interaction of human platelets with live *E*. *coli* O111 focusing in platelet TF-dependent procoagulant activity, thrombin generation, and the contribution of platelet TLR4 in these processes.

## Materials and methods

### Blood collection and platelet preparation

This study was approved by the Institutional Ethics Committee of the Faculty of Medicine, P. Universidad Católica de Chile, Protocol N° 12–202 (http://facultadmedicina.uc.cl/comite/comite.html), and conducted according to principles of the Declaration of Helsinki. All the participating subjects signed an informed consent.

The recruited volunteers were between 19 and 65 years of age with an average of 30 years and a median of 33, 65% of them were women. All the donors had negative results for C-reactive protein and normal values of platelet count and function, measured by platelet aggregation and secretion.

Peripheral venous blood was drawn with minimal or no stasis from healthy adult volunteers not taking antiplatelet drugs during the previous 7 days. Platelet-rich plasma (PRP) and washed, leukocyte-free platelets (L-FP) were processed as previously described [29]. All preparations of L-FP contained less than 1 leukocyte 10^−6^ platelets, assessed by nuclei staining with propidium iodide and flow cytometry, using the Phycoerythrin Conjugated Monoclonal Antibodies (MoAb) Anti-CD45 and Anti-CD14 (BD Biosciences Cat# 555483 and 557154 respectively, San José, CA).

### Bacterial growth and platelet stimulation conditions

In this study we used Enterohemorrhagic *E*. *coli* O111 isolated from a child with acute diarrhea, and identified by multiplex PCR [31]. Nonpathogenic *E*. *coli* DH5α strain was used as an unrelated control strain.

To keep the strains unaltered, a pure culture of the microorganism was stored at -80°C in microbial cryopreservation system Cryobank (Mast Group Ltd.,Liverpool, UK) containing beads with adhered bacteria. One frozen bead with bacteria was incubated in Luria Broth (LB) medium containing 1% Peptone, 0.5% yeast extract and 1% NaCl, pH: 7.0(Merck Millipore, Darmstadt, Germany) and grown overnight at 37°C and 180 rpm in an orbital shaker. Then, fresh LB medium was added (inoculum 1:50) and incubated until attaining mid-exponential phase (OD600_nm_∼0.3, 3.1x10^8^ ufc mL^-1^). After washing twice with PBS the bacteria were centrifuged and subsequently suspended in PRP or washed L-FP. Platelets count was assessed by phase microscopy using a Neubauer chamber in whole blood (WB) and in a Z1 Coulter Counter (Beckman Coulter Inc, USA).

In order to establish the ratio platelet/bacteria to study in this work, diverse proportions were assayed (100/1, 10/1, 1/1, 1/10 and 1/100), choosing the ratio that induced greater platelet activation and procoagulant activity. In the same way, several stimulation times were tested, selecting the period in which platelets without agonist did not show signs of activation but the strongest activation with the bacteria.

Unless otherwise specified, platelets were incubated with living microorganism in a ratio of 1/10 for 30 minutes at 37°C under gentle agitation, in a volume not greater than 500μL. Non-stimulated platelets (N-S) in PRP or L-FP were treated similarly but without agonist, to exclude the effect of temperature and agitation. Each assay was done with different donors, and “n” correspond to the number of volunteers in each assay.

Contribution of platelet TLR4 was studied preincubating the platelets for 15 min with 10 μg mL^-1^ of the inhibitory antibody αTLR4 (InvivoGen Cat# MAb-hTLR4, San Diego, CA),containing FcR Blocking Reagent (Miltenyi Biotec GmbH, Germany) before incubation with bacteria to inhibit unwanted binding of antibodies to human FcγRIIa receptor. The validation of the effectiveness of the FcR blocking reagent (Miltenyi) was done by the test for heparin-induced thrombocytopenia (HIT) antibody [32,33].

To show the absence of interaction of αTLR4 with unspecific bacteria, platelets were stimulated with *E*. *coli* DH5α under the same conditions.

### Protein exposure on platelet surface

The effect of *E*. *coli* O111 or LPS on platelet stimulation was studied in PRP by flow cytometry (Accuri C6 Cytometer; Beckton Dickinson, BD Biosciences, San Jose, CA), using Anti-CD61 MoAb, FITC Conjugated (αCD61-FITC, BD Biosciences Cat# 555753, San Jose, CA) to select platelet population. The fraction of platelets expressing P selectin was obtained using Anti-CD62P MoAb, Phycoerythrin Conjugated (CD62P-PE, BD Biosciences, San Jose, CA) and comparing stimulated versus N-S platelets from a single donor. PRP was incubated with and without *E*. *coli* or LPS at 37°C for 30 minutes under soft agitation. The Mouse IgG1, Isotype Control, Phycoerythrin Conjugated (PE mouse IgG1, BD Biosciences Cat# 555749), was used as negative control.

### Thrombin generation in PRP induced by platelet TF

The platelet potential to generate thrombin *in vitro* after stimulation with *E*. *coli* was measured by a modified Calibrated Automated Thrombogram (CAT) in PRP. Shortly, PRP was incubated with strain O111 in a platelet/bacteria ratio 1/10 for 30 min at 37°C. Then, a 100 μL subsample was dispensed into microtiter plate, adding thrombin fluorescent substrate Z-Gly-Gly-Arg-AMC in Hepes-saline buffer (20μL, pH 7.4 containing 0.1M CaCl_2_) [34]. Neither TF nor phospholipids were added to the reaction mixture. The fluorescence was continuously recorded (1hour, 37°C, under agitation) in a Fluoroskan Ascent FL Microplate Fluorometer (Waltham, MA). The measurements were calibrated with thrombin-μ_2_-macroglobulin complex (Thrombin Calibrator, Stago, Asnières sur Seine, France). The Thrombinoscope^®^ Stago software was used to calculate the amount of generated thrombin. PRP stimulated with ristocetin 1.2mg mL^-1^ and incubated under the same conditions as samples, was used as positive control.

### Platelet TF-dependent activation of factor X

Platelet dependent TF procoagulant activity was measured by FXa generation in L-FP, precluding the potential contribution of leukocyte-derived TF or phospholipids. Platelets were stimulated with *E*. *coli* O111 and compared with aliquots identically treated, but without addition of bacteria (N-S). The test was performed as described [29] with slight modifications. Briefly, 1x10^7^ platelets were added in a microtiter plate, containing 90μL of Tyrode buffer, pH 7.4 (NaCl 121mM, KCl 5mM, MgCl_2_ 0,5mM, CaCl_2_ 1mM, NaHCO_3_ 25mM, NaHPO_4_ 0,4mM, BSA 1%, glucose 5,5mM, pH 7,4) and *E*. *coli* O111 in a platelet/bacteria ratio of 1/10. Then,20μL of each FVIIa (1U mL^-1^), FX (1U mL^-1^), 25mM CaCl_2_ and 40μL of FXa chromogenic substrate were added. Absorbance at 405_nm_ was recorded during 50 min, under agitation at 37°C. The maximal absorbance minus the absorbance at 620_nm_ was expressed as the maximal concentration of FXa, read in a calibration curve built with Purified Human Factor Xa (Aniara Diagnostica, West Chester, OH) and expressed as nmol1x10^-7^platelets. To confirm the specificity of the reaction, inhibitory experiments were carried out pre-incubating the platelets (5min, RT°) with either 100nM Tissue Factor Pathway Inhibitor TFPI (Human TFPI, ADG49B, American Diagnostica GmbH, Pfungstadt Germany), 20 μg mL^-1^ of inhibitory Anti-Human Tissue Factor MoAb (αTF MoAb, American Diagnostica, Cat# 4509) or 20μg mL^-1^ of αGP1bα (inhibitory antibody, clone AP1, Dr. R.R. Montgomery, Milwaukee, WI) before treatment with bacteria. Purified IgG (Sigma-Aldrich Co. Saint Louis, MO) was used to control the specificity of the α-TF MoAb. FXa generation by L-FPs stimulated with either 1IU mL^-1^ of VWF+1.2 mg mL^-1^ Ristocetin (VWF+Ris, Am Biochem & Pharmacy Ltd., London, UK) or 10μM TRAP (Bachem, Torrance, CA) [35] were used as controls of TF-PCA.

### Statistical analysis

Data were analyzed using GraphPad Prism 5.00 (GraphPad Software, San Diego, CA), and presented as mean± SE or median (range). Two-tailed paired t-test and Wilcoxon matched paired test were used for data analysis of normal and non-normal data distribution, respectively. P values<0.05 were considered statistically significant.

## Results

### *E*. *coli* O111 induces platelet activation

**Fig 1:**
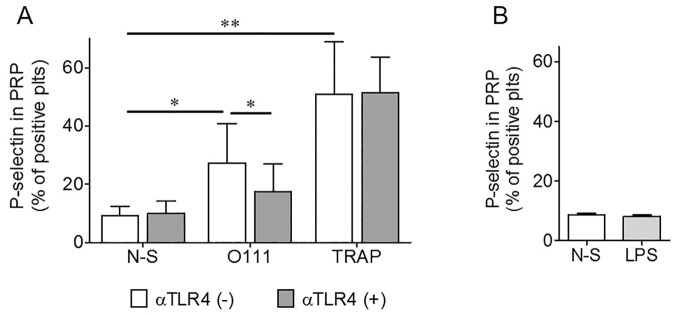
P-selectin expression in platelets stimulated by *E*. *coli* O111. Human platelets were activated with *E*. *coli* for 30 min at 37°C, in a ratio 1:10 (platelet: bacteria). The CD62P expression induced by the strain O111 was significantly higher compared with non-stimulated platelets (*p = 0.03, n = 6), but lower than the response to TRAP (**p = 0.004, n = 5). Previous inhibition of platelet TLR4, reduced significantly the exposure of P-selectin induced by the bacteria (*p = 0.03, n = 6). TLR4 inhibition had no effect on P-selectin expression in TRAP-stimulated platelets (A). Platelets in PRP, stimulated with LPS (10μg/mL) did not show signs of activation (n = 5) (B). The data was analyzed by Wilcoxon signed rank test, two-tailed.

Fig 1A shows a higher P-selectin expression in platelets stimulated with *E*. *coli* O111 for 30 min, compared with N-S platelets (27% [13–40] vs 9.5% [5–12]), respectively. TRAP, a strong platelet agonist, induced faster and higher CD62P expression (±50% positive platelets).

This response to *E*. *coli* O111 was significantly decreased by pre-incubation of PRP with the inhibitory polyclonal α-TLR4 (27% [13–40] to 16.9% [8.8–27]), respectively, (*p 0.03, n = 6). Regardless of the great variation on basal levels of this receptor on N-S platelets, a significant increase of TLR4 was observed in platelets after 30 minutes of interaction with *E*. *coli* from 16.5± 2.9 to 25.73± 2.9, respectively (n = 7, ***p 0.0007, paired T test, not shown). In contrast to *E*. *coli*, the effect of TRAP on P-selectin exposure was not significantly modified by TLR4 inhibition (Fig 1A).

On the other hand, platelet stimulation with ultrapure *E*. *coli* O111-LPS (Invivogen San Diego, CA), tested at three concentrations (5, 10 and 50 μg/ml) did not increase platelet P-selectin expression. Representative results obtained using 10 μg/ml of LPS are shown in Fig 1B.

### *E*. *Coli* O111 accelerates platelet-TF dependent thrombin generation in PRP. Contribution of TLR4

**Fig 2:**
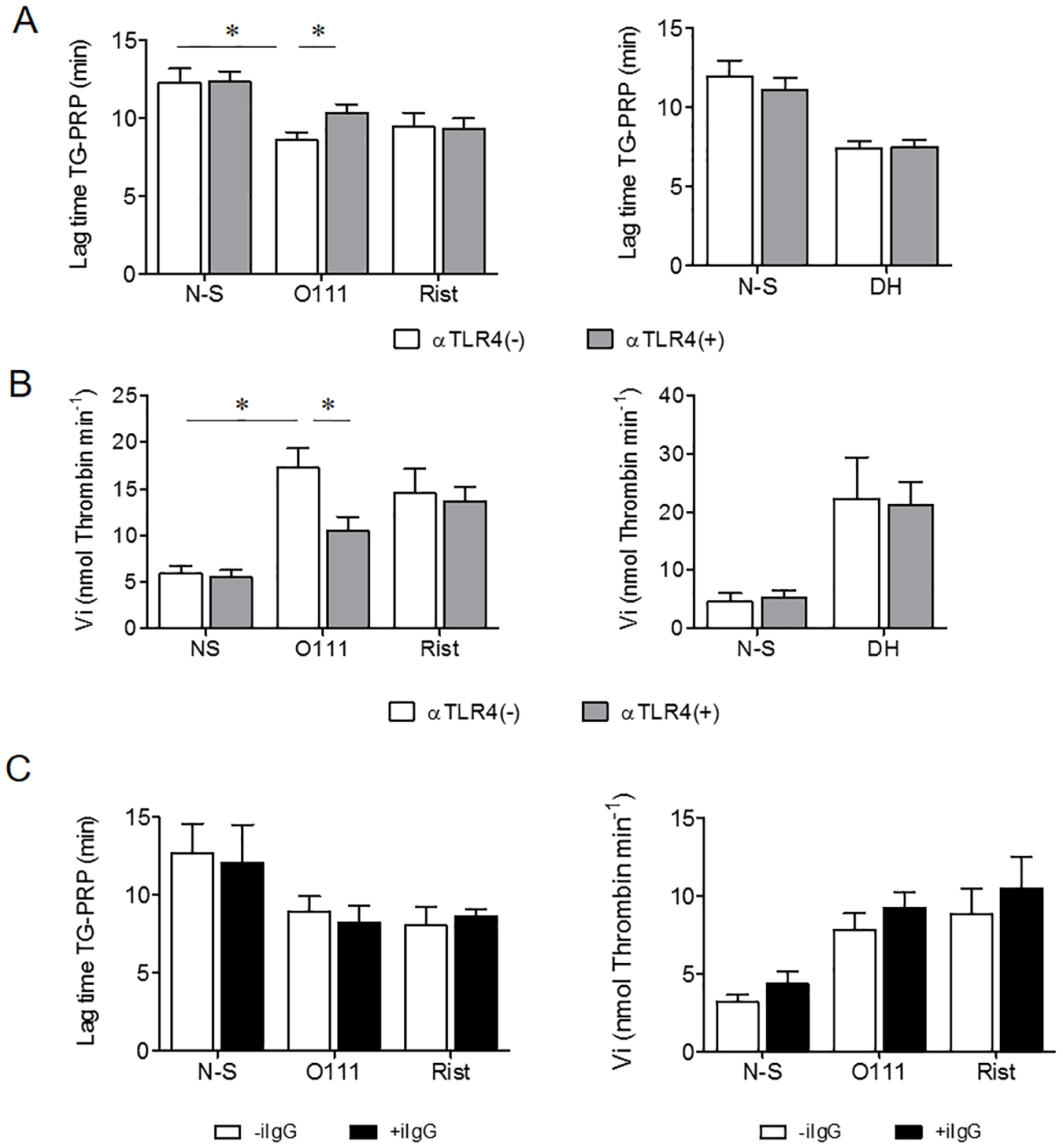
*E*. *coli* O111 induces platelet TF-dependent thrombin generation, involving TLR4 activation. This thrombin generation assay did not use an external source of TF and phospholipids, and FcγRIIA was previously blocked with FcR blocking reagent. The interaction of platelets with *E*. *coli* O111 shortened the Lag Time (*p < 0.016, n = 7) (A) and increased the Velocity index of TG-PRP (* p< 0.016, n = 7) (B) compared with N-S platelets. This effect was similar to that obtained after PRP stimulation with ristocetin (used as positive control) or the unrelated strain *E*. *coli* DH5α, which also shortened Lag Time (* p <0.01, n = 3) and increased the Velocity index of TG-PRP (* p < 0.03, n = 3) (right panels figures A and B). TLR4 inhibition (along with FcγR previously blocked) prolonged the Lag Time (*p = 0.016, n = 7) and reduced the Velocity Index (*p = 0.016, n = 7) of TG in PRP incubated with the strain O111. Nevertheless, this inhibition did not have effect in Lag Time nor Velocity Index when PRP was stimulated with ristocetin or the strain DH5α (right panels figures A and B). Figure 2C shows no significant changes in TG-PRP generated by the strain O111 when a non-immune IgG was used (n = 4). The data was analyzed by Wilcoxon signed rank test, two-tailed.

PRP clotting was induced without exogenous TF and phospholipids, and FcγRIIA was previously blocked with FcR blocking reagent as described in Methods. Fig 2A shows that platelet interaction with O111 strain shortened significantly the time to thrombin burst (lag time) to 8.59±0.5 min from 12.3±0.9 min in control platelets (*p <0.016, n = 7). Similarly, the velocity index (e.g., the effective rate of thrombin generation between lag time and time to peak), was significantly higher in *E*. *coli* stimulated PRP than in N-S platelets (17.3± 2.0 vs 5.8±0.8 nmol thrombin min^-1^), respectively (*p<0.016, n = 7) (Fig 2B).

The response to *E*. *coli* O111 was inhibited by pre-incubating PRP with αTLR4. Fig 2A and 2B (left panels) show a significant prolongation of lag time (8.59± 0.5 to 10.4±0.5 min, * p = 0.016, n = 7) and decrease of velocity index (17.3± 2.0 to 10.5± 1.5, nmol thrombin min^-1^, * p = 0.016, n = 7), compared with non-inhibited platelets. The right panels of Fig 2A and 2B show the results obtained in TG-PRP with platelets stimulated with a different strain of *E*. *coli* (DH5α). Although the strain did induced changes on both parameters of TG-PRP, they were independent of TLR4, since its inhibition did not increased Lag time, neither shortened Vi. Pre-incubation of platelets with a non-immune mice IgG showed no significant changes in the TG-PRP generated after stimulation with either bacteria or Rist (n = 4) (Fig 2C).

### Bacterial stimulation of platelets triggers TF-dependent PCA involvingTLR4

**Fig 3:**
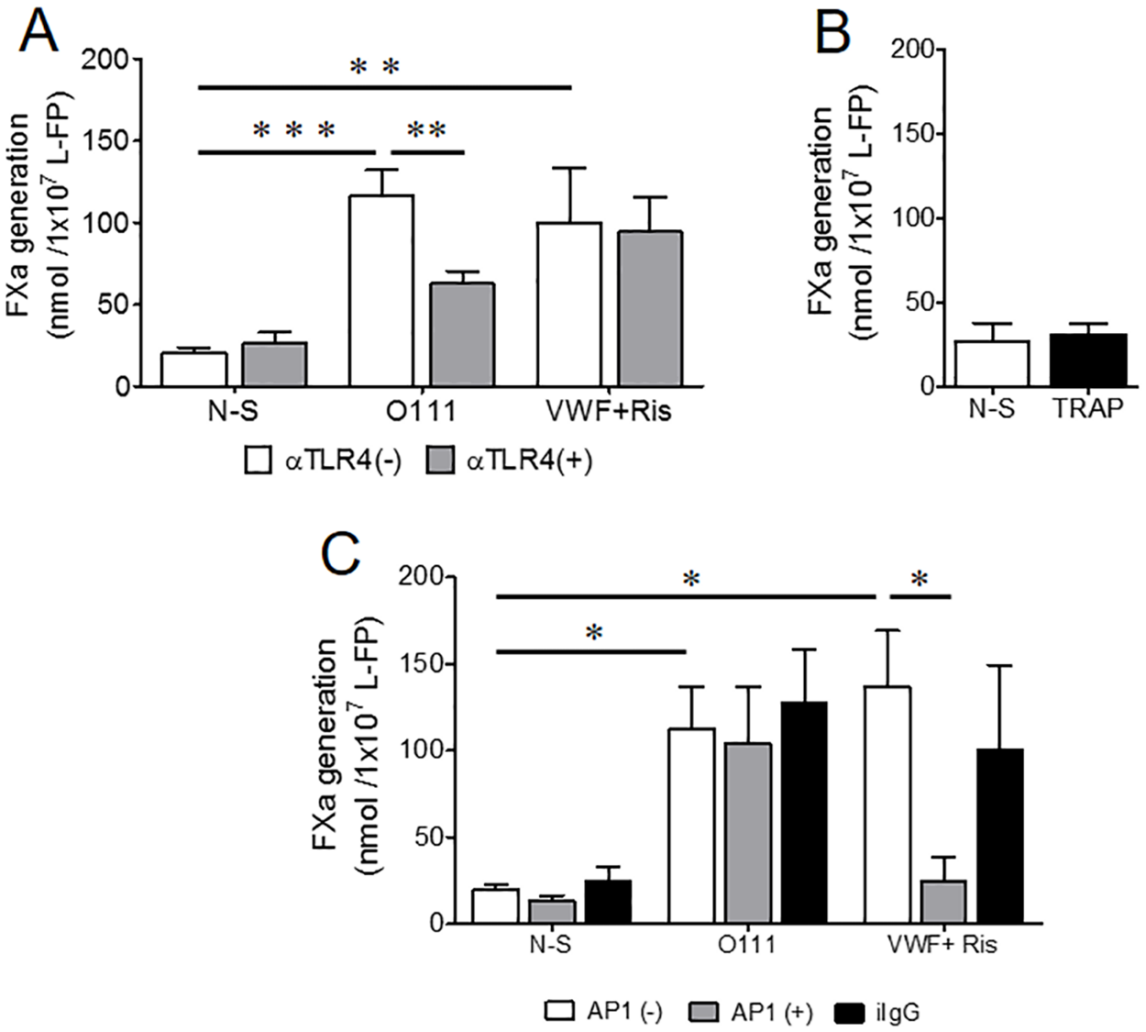
*E*. *coli* O111 induced platelet-dependent TF procoagulant activity, involving TLR4 activation. *E*. *coli* O111 increased FXa generation compared to N-S platelets (***p = 0.0005, n = 8) in assays without external source of TF and phospholipids. The amount of FXa generated was not significantly different than that of platelets stimulated with VWF+Ris (**p = 0.004, n = 8). TLR4 inhibition resulted in a ±-50% reduction in the generated FXa with strain O111 (**p = 0.004, n = 8), but did not influence the FXa generated by VWF+Ris activation of platelets (A). TRAP stimulated platelets did not generated FXa (B). Data analyzed by Paired t test, two-tailed. Pre-incubation of platelets with an inhibitory α-GPIbα MoAb (AP1) did not affect the FXa generated by *E*. *coli* but instead, reduce significantly the FXa triggered in platelets with VWF+Ris (*p = 0.03, n = 6) (C). Pre-incubation of platelets with a non-immune irrelevant IgG did not induced significant changes in FXa generation when stimulated with bacteria or VWF+Ris (n = 4) (C). Data were analyzed with Wilcoxon signed rank test, two tails.

*E*. *coli* O111 activation increased by nearly six-fold the FXa generated by N-S platelets: 116±15 vs 21± 3 nmol FXa 1x10^-7^ platelets (Fig 3A). This enhancement is similar to that obtained in control experiments with L-FPs stimulated with VWF+Ris (99±38 nmol FXa 1x10^-7^ platelets). In contrast, TRAP stimulation did not induce FXa enhancement in L-FPs (Fig 3B).

Inhibition of TLR4 in L-FPs stimulated with *E*. *coli* reduced by nearly 50% the FXa generation, from 116±15 to 62 ± 7 nmol FXa 1x10^-7^ platelets (Fig 3A), whereas this effect was not observed in VWF+Ris stimulated platelets. In contrast, pre-incubation of L-FPs with AP1, an inhibitory α-GPIbαMoAb, dampened FXa generated by VWF+Ris activation (*p 0.03, n = 6) whereas this antibody had no inhibitory effect on the procoagulant activity induced by *E*. *coli*, Fig 3C. Pre-incubation of platelets with a non-immune irrelevant IgG did not induced significant changes in FXa generation when stimulated with bacteria or Rist (n = 4) (Fig 3C).

**Fig 4:**
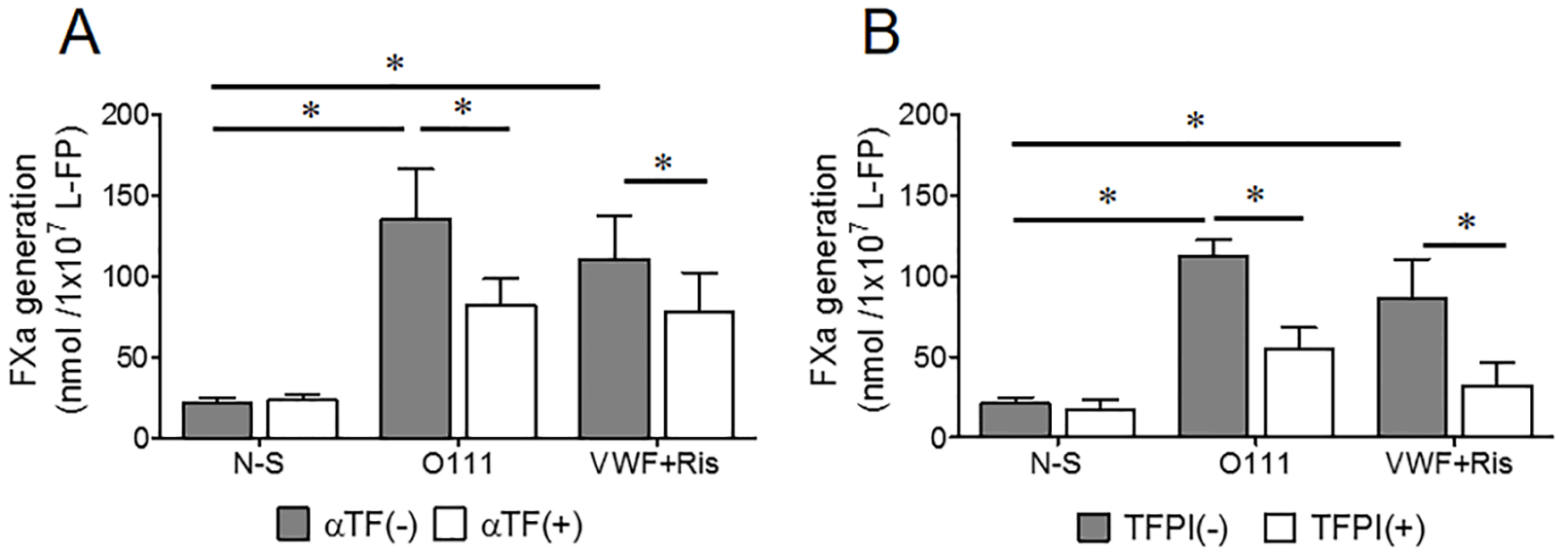
Inhibition of platelet procoagulant activity induced by *E*. *coli* O111 by αTF MoAb and TFPI. Leukocyte-free platelets (L-FP) were pre-incubated with either αTF MoAb or TFPI, before stimulation with *E*. *coli* O111 or VWF+Ris (positive control). Inhibition of TF reduce the generation of FXa by the strain O111 around 40% (*p = 0.031, n = 6) and, when platelets were activated with VWF+Ris, the inhibition was about 30% (*p = 0.031, n = 5) (A). Data analyzed with Wilcoxon signed rank test, two tails. Similar results were observed in L-FPs incubated with TFPI before the stimulation with *E*. *coli* or VWF+Ris, reaching levels of inhibition of 50% (*p 0.03, n = 4) and 60% (*p 0.04, n = 5), respectively (B). The data were analyzed by Mann-Whitney test.

The inhibitory α-TF MoAb decreased the TF-dependent PCA elicited by *E*. *coli* O111 from 136±30 to 82 ± 16 nmol FXa 1x10^-7^ platelets (*p = 0.031, n = 6, Fig 4A). Likewise, preincubation with TFPI diminished by nearly 50% the FXa generation induced by the bacteria, from 112±10 to 55 ± 12 nmol FXa 1x10^-7^ platelets (*p = 0.03, n = 4, Fig 4B). Inhibition of TF with α-TF or TFPI also dampened the FXa generated by L-FPs activated with VWF+Ris in 30 and 62%, respectively (Fig 4A and 4B).

## Discussion

Platelets play a role in innate immunity and defense against pathogens, but reports on their procoagulant response triggered by Gram-negative bacteria are scarce. The consequences of platelet-bacteria interaction are determined among others factors, by type of bacterial strains, the ratio platelet/bacteria and also by an important inter-subject variability of platelet responses [16,36]. The aim of this study was to explore the *ex vivo* interaction of human platelets and live *E*. *coli* O111 strain, focusing in platelet procoagulant activity and TLR4 role in this response and thus, to better understand the role of platelets in the hemostatic abnormalities of systemic infections.

The Enterohemorrhagic *E*. *coli* strain O111 induced platelet activation with α-granules secretion, assessed by membrane P-selectin expression in PRP platelets. However, ultrapure O111-LPS as agonist did not induced platelet activation (evaluated with three concentrations), even when tested along with subthreshold concentrations of classical agonists such as ADP (not shown).

Considering that the platelet response to LPS is highly heterogeneous [17–19,27,37,38], we believe that this result it is not unexpected, since they are also in agreement with the results of Moriarty *et al*, who showed that EHEC serotype O157 induced platelet aggregation, but pure LPS from the same strain did not [39].

TLR4 involvement in the *E*. *coli* O111-induced platelet activation is supported by the fact that P-selectin expression did not vary in platelets of PRP in which FcγRIIa was blocked but, it was diminished when platelet’s TLR4 was neutralized. Therefore, under our experimental approach, platelet activation induced by EHEC O111 involves TLR4, without FcR engagement. Our results add evidence to the role of TLR4 in platelet activation, showed in studies with TLR4 deletion in mice and the important contribution of this receptor in platelet activation in hemorrhagic shock [40].

The platelet-bacteria interaction is highly variable, mainly due to the strain studied and the function analyzed. For example, *E*. *coli* O18 does not induce CD62P expression on platelets and *E*. *coli* K12 induces a strong exposure of this protein, but independent of TLR4 activation [41].

Our findings are also not in contradiction to previous published works, where other *E*. *coli* strains induced platelet activation through FcγRIIA without TLR4 participation, since they were done with pathotypes of *E*. *coli* different from EHEC O111, as the neuropathogenic and uropathogenic *E*. *coli* strains RS218 and CFT073, respectively [42]. Furthermore, they evaluated platelet aggregation as activation marker[42] as in the work of Moriarty *et*. *al*. with the EHEC O157 [39]. Here, we assessed the effectiveness of EHEC O111 to elicit the procoagulant activity of platelets and evaluated the participation of their own TF and TLR4 on it.

Several reports, including ours, have provided accumulating evidence that megakaryocytes and platelets synthesize TF able to trigger clotting upon specific platelet stimulation [27–29,35,43]. In this regard, Rondina *et al* [30] showed that the septic *milieu* triggers the expression of spliced TF mRNA by circulating platelets and that TLR4 signaling inhibition blocked this splicing in platelets stimulated with LPS. We found that *E*. *coli* O111 strain did induce platelet TF-dependent PCA, measured by both, FX activation in washed L-FPs and thrombin generation in PRP platelets. Moreover, the amount of FXa generated by this bacterial strain was equivalent to that induced by direct GPIbα activation with VWF+Ris, which we described as a better inducer of platelet TF activation [35] than other potent agonists, like TRAP and collagen. These results confirm that TF is originated from platelets, since neither exogenous TF nor phospholipids were present in the reaction. Moreover, FXa generation induced by either *E*. *coli* O111 or by VWF+Ris was inhibited by both, blocking α-TF MoAb and TFPI. Other reports assign an important role to TF-bearing microparticles in the hemostatic disorders of systemic infections. Interestingly, most circulating microparticles also express platelet antigens [44,45], suggesting again a platelet origin of this TF.

The effect of *E*. *coli* was mediated by TLR4 activation, as demonstrated by the inhibition of PCA response by an α-TLR4. This antibody had no effect on the TF activation elicited by VWF+Ris. In contrast, inhibition of GPIbα with AP1 MoAb dampened the FXa generated by VWF+Ris stimulation, but had null effect on FXa induced *E*. *coli* through TLR4 complex activation, emphasizing the specificity of this bacterial activation pathway. Of note, the response to *E*. *coli* O111 required the living bacteria, since ultra-pure *E coli* O111 LPS did not generate FXa in platelets. The lack of effect of LPS does not disagree with Rondina *et al*. findings described above [30], since these authors did not used intact platelets to measure TF PCA instead, they used isolated membranes or microparticles from platelets previously incubated with LPS during 120 minutes.

In platelets, *E*. *coli* O111 also accelerated the thrombin generation, measured by both the lag time and the velocity index of the reaction, and this response was also retarded by αTLR4, which suggest that in the hemostatic derangement caused by this strain, platelet TLR4 might be involved.

The current concept of physiologic cell-based clotting system assigns the platelets a pivotal role in assembling the coagulation complexes on their membranes and their contribution as “TF-bearing cells”, to trigger the coagulation reactions [28]. In systemic infections, platelets would also play a key pathophysiological role as procoagulant effectors. In this context, the diversity in the procoagulant response between individuals could be related to the physiological variety in expression, synthesis or activity of platelet-TF, as well as TLR4 expression pattern.

The sum of our results supports a major role of TLR4 in the procoagulant response of platelets to EHEC O111, however, they cannot be extended to other gram-negative bacterial strains or rule out the contribution of other platelet receptors not assessed in this study. Nevertheless, our experimental approach could be used as template for studying the procoagulant response of platelets to other microorganisms.

Despite these limitations, we here unveil a novel bacteria-induced activation of platelet TF *in vitro*, involving TLR4 pathway. Additional studies are needed to know if our results mimic some *in vivo* condition and whether TLR4 could become a new target to modulate the hemostatic disorder of sepsis.

Lastly, we believe that our findings will provide a better understanding of the role of platelet-bacteria interaction in hemostatic abnormalities of sepsis and lay a foundation for the development of improved strategies for managing the prothrombotic state of septic patients, aiming to achieve a satisfactory outcome.
